# Supplementary material for: Sub-centrosomal mapping identifies augmin-γTuRC as part of a centriole-stabilizing scaffold
Source: Nat Commun. 2021 Oct 15;12:6042. doi: 10.1038/s41467-021-26252-5 (PMC8519919; doi:10.1038/s41467-021-26252-5)
Supplement: Supplementary file 1 — Supplementary Information [file 41467_2021_26252_MOESM1_ESM.pdf]

Supplementary information to

Sub-centrosomal mapping identifies augmin-γTuRC as part of a centriole-stabilizing scaffold

Schweizer et al.

Content:

Supplementary figures 1-5

Supplementary table 1

a

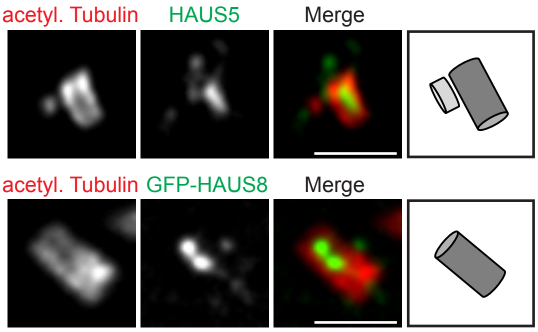
acetyl. Tubulin
HAUS5
Merge
acetyl. Tubulin
GFP-HAUS8
Merge

b

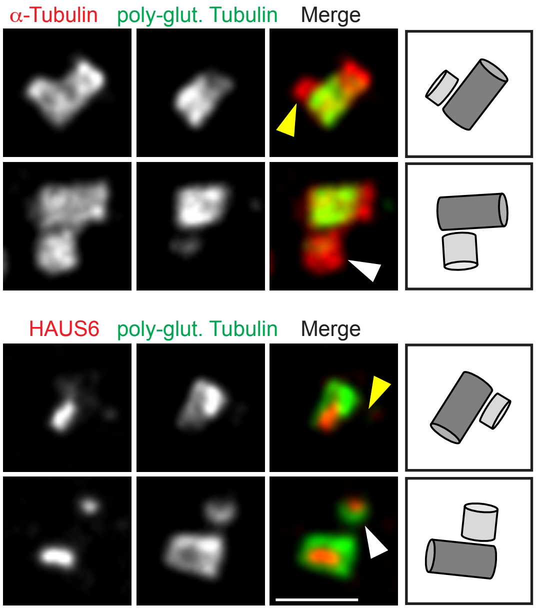
α-Tubulin
poly-glut. Tubulin
Merge
HAUS6
poly-glut. Tubulin
Merge

Supplementary Figure 1 Augmin localizes to the centriole lumen late during the cell cycle. (**a**) Centrioles of parental U2OS cells or U2OS cells stably expressing EGFP-HAUS8 in ExM stained for acetylated α-tubulin and HAUS5 or GFP (EGFP-HAUS8). (**b**) Centrioles of U2OS cells in ExM stained for α-tubulin and poly-glutamylated tubulin or HAUS6 and poly-glutamylated tubulin. Yellow arrowheads point to daughter centrioles that lack HAUS6/poly-glutamylation, white arrowheads point to poly-glutamylated daughter centrioles (that have HAUS6 in the lumen). Scale bar (all panels), 2 µm. Cartoons illustrate configurations of mother (dark grey) and daughter (light grey) centrioles.

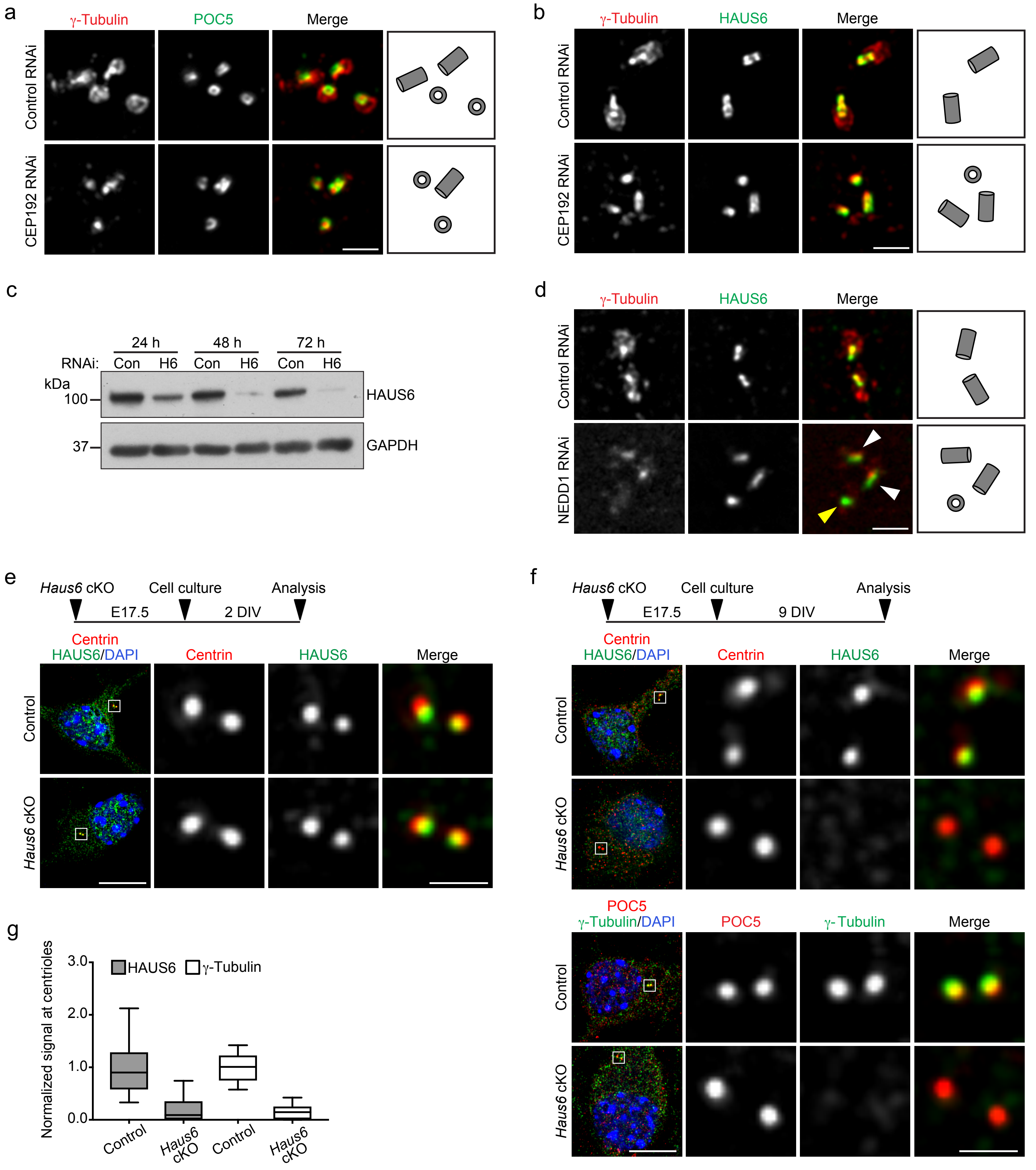

a
γ-Tubulin
POC5
Merge
Control RNAi
CEP192 RNAi
b
γ-Tubulin
HAUS6
Merge
Control RNAi
CEP192 RNAi
c
24 h
48 h
72 h
RNAi:
Con
H6
Con
H6
Con
H6
kDa
100
37
HAUS6
GAPDH
d
γ-Tubulin
HAUS6
Merge
Control RNAi
NEDD1 RNAi
e
Haus6 cKO
Cell culture
Analysis
E17.5
2 DIV
Centrin
HAUS6/DAPI
Centrin
HAUS6
Merge
Control
Haus6 cKO
f
Haus6 cKO
Cell culture
Analysis
E17.5
9 DIV
Centrin
HAUS6/DAPI
Centrin
HAUS6
Merge
Control
Haus6 cKO
POC5
γ-Tubulin/DAPI
POC5
γ-Tubulin
Merge
Control
Haus6 cKO
g
Normalized signal at centrioles
3.0
2.0
1.0
0.0
HAUS6
γ-Tubulin
Control
Haus6 cKO
Control
Haus6 cKO

Supplementary Figure 2 γTuRC centriole localization depends on CEP192 and augmin. (**a**) Centrioles of control and CEP192 RNAi U2OS cells in U-ExM stained for γ-tubulin and POC5. (**b**) Centrioles of control and CEP192 RNAi U2OS cells in U-ExM stained for γ-tubulin and HAUS6. (**c**) Western Blot analysis of HAUS6 in extracts from control (Con) and HAUS6 RNAi (H6) U2OS cells at different time points after siRNA transfection. GAPDH was used as loading control. (**d**) Centrioles of control and NEDD1 RNAi U2OS cells in ExM stained for γ-tubulin and HAUS6. White arrowheads point to centrioles that have γ-tubulin in the lumen, yellow arrowhead points to a centriole that is depleted of luminal γ-tubulin, but has HAUS6 in the lumen. Scale bar (**a**,**b**,**d**), 2 µm. Cartoons illustrate centriole configurations. (**e**) Murine control and *Haus6* cKO neurons, dissected from E17.5 embryos and cultured for 2 days (2 DIV) as depicted in the schematic, were stained for centrin, HAUS6 and DNA. (**f**) Murine control and *Haus6* cKO neurons, dissected from E17.5 embryos and cultured for 9 days (9 DIV) as depicted in the schematic, were stained for centrin, HAUS6 and DNA, or for POC5, γ-tubulin and DNA. Scale bar in (**e**) and (**f**), 10 µm (cells) or 1 µm (magnified centrioles). (**g**) Relative fluorescence signals for HAUS6 and γ-tubulin at centrioles in control and *Haus6* cKO neurons at 9 DIV. The box plot contains pooled data from 2 independent experiments, 16-20 centrioles analyzed per condition and experiment. Boxes extend from the 25th to the 75th percentile, the horizontal line indicates the median, whiskers depict the 10th and 90th percentiles. Source data are provided as a Source Data file.

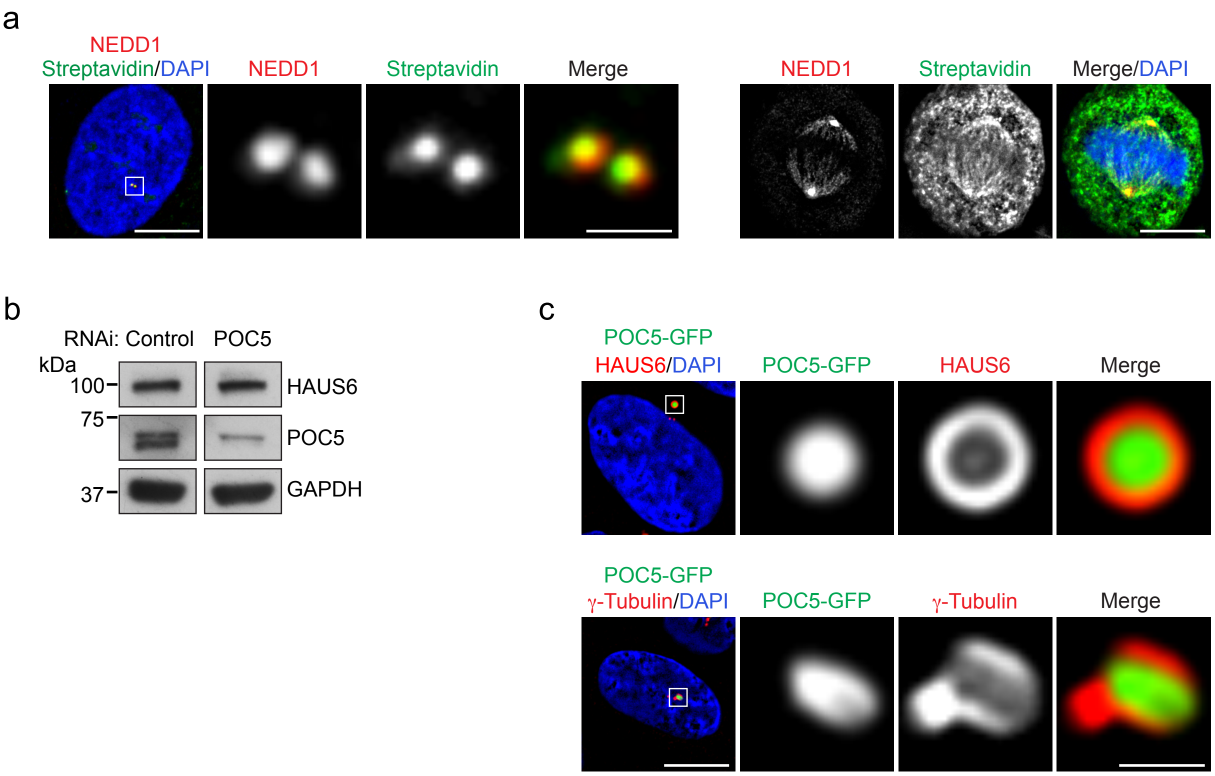
a
NEDD1
Streptavidin/DAPI
NEDD1
Streptavidin
Merge
NEDD1
Streptavidin
Merge/DAPI
b
RNAi: Control
POC5
kDa
100
75
37
HAUS6
POC5
GAPDH
c
POC5-GFP
HAUS6/DAPI
POC5-GFP
HAUS6
Merge
POC5-GFP
γ-Tubulin/DAPI
POC5-GFP
γ-Tubulin
Merge

Supplementary Figure 3 Identification of POC5 as a proximity interactor of HAUS6. (**a**) Interphase or mitotic U2OS cells stably expressing BirA-HAUS6 were incubated with biotin and stained for NEDD1, biotin (568-conjugated streptavidin) and DNA. (**b**) Western Blot analysis of POC5 in extracts from control and POC5 RNAi U2OS cells. GAPDH was used as loading control. (**c**) U2OS cells stably expressing POC5-GFP stained for GFP (POC5-GFP), HAUS6 or γ-tubulin and DNA. Scale bar (all panels), 10 μm (cells) or 1 μm (magnifications).

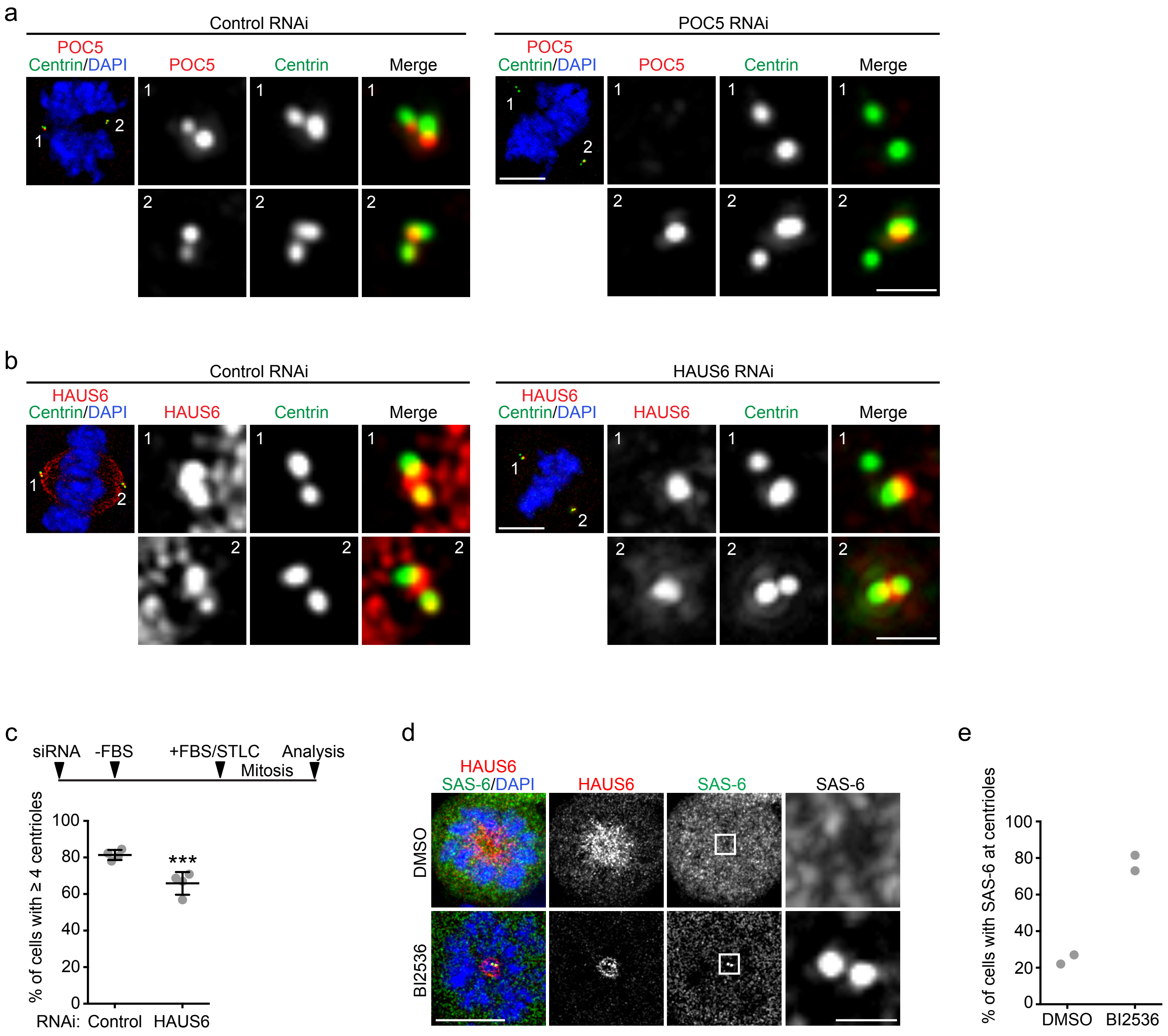
a
Control RNAi
POC5 RNAi
POC5
Centrin/DAPI
POC5
Centrin
Merge
b
HAUS6 RNAi
HAUS6
Centrin/DAPI
HAUS6
c
siRNA
-FBS
+FBS/STLC
Analysis
Mitosis
% of cells with ≥ 4 centrioles
***
RNAi: Control HAUS6
d
HAUS6
SAS-6/DAPI
SAS-6
DMSO
BI2536
e
% of cells with SAS-6 at centrioles
DMSO
BI2536

Supplementary Figure 4 Centriole numbers are not reduced in cycling POC5 RNAi or HAUS6 RNAi cells. (**a**) Mitotic control or POC5 RNAi U2OS cells stained for POC5, centrin and DNA. Magnifications show centriole staining at each of the two spindle poles. (**b**) Mitotic control or HAUS6 RNAi U2OS cells stained for HAUS6, centrin and DNA. Magnifications show centriole staining at each of the two spindle poles. (**c**) Quantifications of the percentage of control or HAUS6 RNAi RPE1 cells with ≥ 4 centrioles (centrin foci) after prolonged mitotic arrest following the depicted treatment. Data points are from 4 independent experiments, 100-500 cells analyzed per condition and experiment. The horizontal line depicts the mean, error bars represent standard deviations ($p = 7.5495e-15$, generalized linear model with binomial distribution). (**d**) Control RNAi U2OS cells after 24 h in mitosis without (DMSO) or with (BI2536) PLK1 inhibition, stained for HAUS6, SAS-6 and DNA. (**e**) Quantifications of the percentage of cells as in (**d**) with SAS-6 at centrioles. Data points are from 2 independent experiments, 100-200 cells analyzed per condition and experiment. *** $p < 0.001$. Scale bar (**a,b,d**), 10 µm (cells) or 1 µm (magnifications). Source data are provided as a Source Data file.

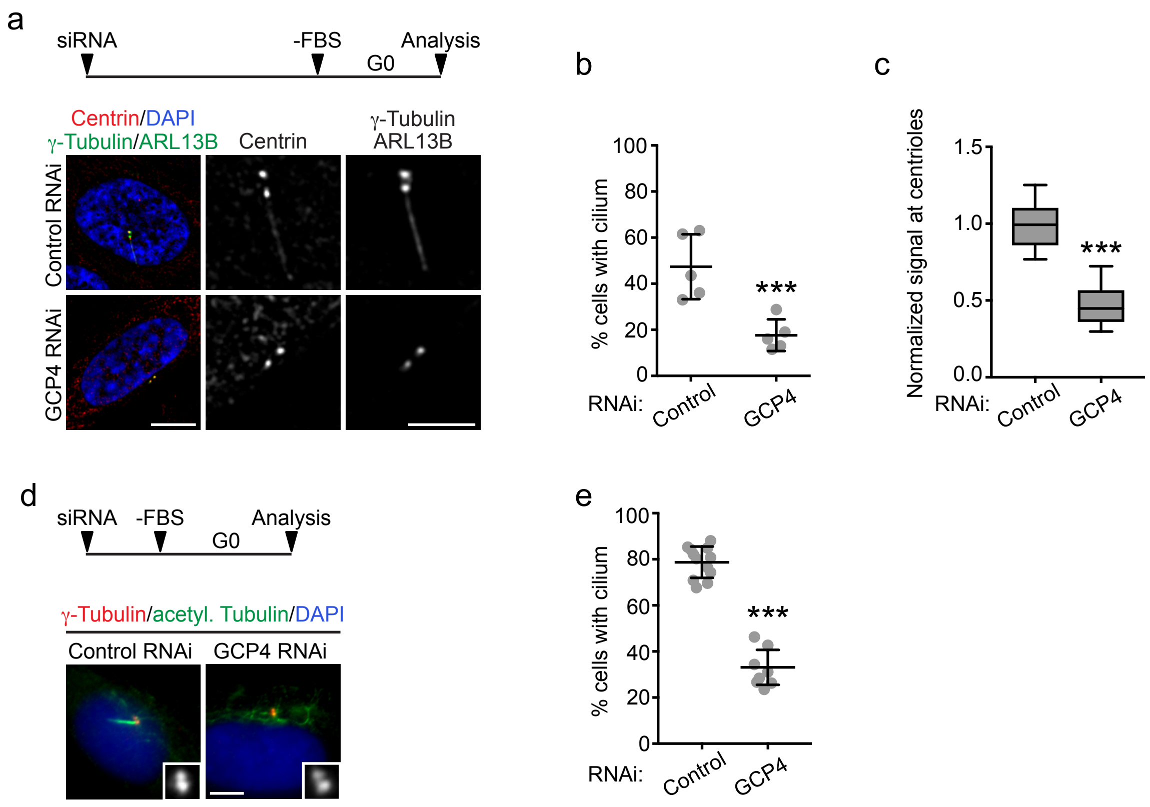
a
siRNA
-FBS
G0
Analysis
Centrin/DAPI
γ-Tubulin/ARL13B
Centrin
γ-Tubulin
ARL13B
Control RNAi
GCP4 RNAi
b
% cells with cilium
***
RNAi:
Control
GCP4
c
Normalized signal at centrioles
***
RNAi:
Control
GCP4
d
siRNA
-FBS
G0
Analysis
γ-Tubulin/acetyl. Tubulin/DAPI
Control RNAi
GCP4 RNAi
e
% cells with cilium
***
RNAi:
Control
GCP4

Supplementary Figure 5 γTuRC is required for ciliogenesis. (**a**) Serum-starved control and GCP4 RNAi RPE1 *p53* KO cells stained for centrin, γ-tubulin, ARL13B and DNA. Scale bar, 10 µm (cells) or 5 µm (magnifications of centrioles/cilia). The experimental design is depicted schematically. (**b**) Quantifications of the percentage of ciliated cells as in (**a**). Data points are from 5 independent experiments, 200-302 cells analyzed per condition and experiment. The horizontal line depicts the mean, error bars represent standard deviations ($p < 2.22e-16$, generalized linear model with binomial distribution). (**c**) Relative fluorescence intensity for γ-tubulin at centrioles in cells as in (**a**). The box plot contains pooled data from 3 independent experiments, 51-56 cells analyzed per condition and experiment. Boxes extend from the 25th to the 75th percentile, the horizontal line indicates the median, whiskers depict the 10th and 90th percentiles ($p < 2.22e-16$, mixed effects linear model). (**d**) Serum-starved control and GCP4 RNAi BJ cells stained for γ-tubulin, acetylated α-tubulin (acetyl. Tubulin) and DNA. Insets show magnifications of centrosomal γ-tubulin staining. The experimental design is depicted schematically. Scale bar, 5 µm (**e**) Quantifications of the percentage of ciliated cells as in (**d**). Data points are from 12 independent experiments, 64-206 cells analyzed per condition and experiment. The horizontal line depicts the mean, error bars represent standard deviations ($p < 2.22e-16$, generalized linear model with binomial distribution). *** $p < 0.001$. Source data are provided as a Source Data file.

Supplementary table 1:

primer name	primer sequence (5’-3’)
mAug6KO_FW	CAACCCGAGCAACAGAAACC
mAug6KO_Rev	CCTCCCACCAACTACAGACC
26994	GCTGGAAGATGGCGATTAGC
30672	TCAGCCTGGTTACAAGGAACA
oIMR7338	CTAGGCCACAGAATTGAAAGATCT
oIMR7339	GTAGGTGGAAATTCTAGCATCATCC
