# Supplementary material for: Sub-centrosomal mapping identifies augmin-γTuRC as part of a centriole-stabilizing scaffold
Source: Nat Commun. 2021 Oct 15;12:6042. doi: 10.1038/s41467-021-26252-5 (PMC8519919; doi:10.1038/s41467-021-26252-5)
Supplement: Supplementary file 4 — Description of additional supplementary files [file 41467_2021_26252_MOESM4_ESM.docx]

Description of additional supplementary information

Title: Supplementary Data 1

Description: Mass spectrometry analysis of proximity interactors of BirA-HAUS6
