# Supplementary material for: Sub-centrosomal mapping identifies augmin-γTuRC as part of a centriole-stabilizing scaffold
Source: Nat Commun. 2021 Oct 15;12:6042. doi: 10.1038/s41467-021-26252-5 (PMC8519919; doi:10.1038/s41467-021-26252-5)
Supplement: Supplementary file 5 — Reporting Summary [file 41467_2021_26252_MOESM5_ESM.pdf]

nature research

Corresponding author(s): Jens Lüders

Last updated by author(s): 30/08/20

Reporting Summary

Nature Research wishes to improve the reproducibility of the work that we publish. This form provides structure for consistency and transparency in reporting. For further information on Nature Research policies, see our Editorial Policies and the Editorial Policy Checklist.

Statistics

For all statistical analyses, confirm that the following items are present in the figure legend, table legend, main text, or Methods section.

n/a	Confirmed	
☐	☒	The exact sample size (*n*) for each experimental group/condition, given as a discrete number and unit of measurement
☐	☒	A statement on whether measurements were taken from distinct samples or whether the same sample was measured repeatedly
☐	☒	The statistical test(s) used AND whether they are one- or two-sided<br>*Only common tests should be described solely by name; describe more complex techniques in the Methods section.*
☒	☐	A description of all covariates tested
☐	☒	A description of any assumptions or corrections, such as tests of normality and adjustment for multiple comparisons
☐	☒	A full description of the statistical parameters including central tendency (e.g. means) or other basic estimates (e.g. regression coefficient) AND variation (e.g. standard deviation) or associated estimates of uncertainty (e.g. confidence intervals)
☐	☒	For null hypothesis testing, the test statistic (e.g. *F*, *t*, *r*) with confidence intervals, effect sizes, degrees of freedom and *P* value noted<br>*Give P values as exact values whenever suitable.*
☒	☐	For Bayesian analysis, information on the choice of priors and Markov chain Monte Carlo settings
☒	☐	For hierarchical and complex designs, identification of the appropriate level for tests and full reporting of outcomes
☒	☐	Estimates of effect sizes (e.g. Cohen's *d*, Pearson's *r*), indicating how they were calculated

Our web collection on statistics for biologists contains articles on many of the points above.

Software and code

Policy information about availability of computer code

Data collection

Centriole/cilia countings were performed on a Leica DMI6000B microscope (1.4 NA 100× oil immersion objective) or an Axiovert 200M (1.4 NA 63× Plan Apo objective). Images were acquired with an Orca AG camera (Hamamatsu) on a Leica DMI6000B microscope equipped with a 1.4 NA 100× HCX Plan Apo oil immersion objective, filter sets L5 (Leica), TX2 (Leica), DAPI ET (F46-900; AHF analysentechnik), and light source EL6000 (Leica). AF6000 software (Leica, 3.0.0 build 81.34) was used for image acquisition and blind deconvolution. Alternatively, images were acquired with an MRm camera (Zeiss) on an Axiovert 200M (Zeiss) using a 1.4 NA 63× Plan Apo oil immersion objective (Zeiss; Fig. 6c and Supplementary Fig. 5d) or a 1.4 NA 100× UPLSApo oil immersion objective (Olympus; Fig. 6f) using Axiovision software (Zeiss: v4.8.2) and the following filters: Ex G 365 nm - Em BP 445/50 nm, Ex BP 475/40 nm - Em BP 530/50 nm, Ex BP 546/12 nm - Em LP 590 nm and light source: HBO 103 W/2. Images were deconvolved using the Huygens software (Scientific Volume Imaging: v16.05). Images presented in Fig. 5a,b were acquired with an Orca-flash4 V2+ CMOS camera (Hamamatsu) on a Leica Dmi8 equipped with a Yokogawa CSU-X1 5000 rpm spinning disc unit and a 1.4 NA 63× HC PL Apo objective, oil (Leica) with a 0.8× lens using Metamorph software (Molecular Devices: v7.8.13)) and the following filters: 488 nm (150 mW) - Em 525/15 nm, 561 nm (100 mW), Em 607/18 nm and light source: 4 colours ILE laser diode (Andor technology). Images were deconvolved using the Huygens software (Scientific Volume Imaging v16.05). For super-resolution microscopy, we imaged cells using fast random illumination microscopy (RIM) with a binary phase optical modulator (QXGA SLM Fourth Dimension) using a 100× objective lens (CFI SR APO 100XH ON 1,49 DT 0,12 Nikon) mounted on a Nikon Eclipse Ti-E and two Hamamatsu SCMOS cameras (ORCA FLASH FUSION) aligned for two colour imaging. A series of 400 3D Speckle illuminations was made on the object plane thanks to the random phase modulation from the SLM conjugate with the pupil plane of the microscope objective. A 200 nm Z interval was acquired on each image. Super-resolution images were processed by a variance matching process using random speckle illuminations with wiener parameter equal to 0.008 and 35 iterations (AlgoRIM software v1.1, https://github.com/teamRIM/tutoRIM)).

Data analysis	Images were processed in ImageJ (2.0.0-rc-43/1.51a) and Photoshop (version 11.0). Quantification of fluorescence intensities and length measurements were performed in ImageJ (2.0.0-rc-43/1.51a). Statistical tests were performed with 'R' version 4.0.5 (2021-03-31) using the packages lme4_1.1-26, lmerTest_3.1-3, blme_1.0-5, multcomp_1.4-17, MASS_7.3-54.

For manuscripts utilizing custom algorithms or software that are central to the research but not yet described in published literature, software must be made available to editors and reviewers. We strongly encourage code deposition in a community repository (e.g. GitHub). See the Nature Research guidelines for submitting code & software for further information.

Data

Policy information about availability of data

All manuscripts must include a data availability statement. This statement should provide the following information, where applicable:

- Accession codes, unique identifiers, or web links for publicly available datasets
- A list of figures that have associated raw data
- A description of any restrictions on data availability

All source data associated with the presented results of this study are provided in Source Data file. The mass spectrometry proteomics data have been deposited to the ProteomeXchange Consortium via the PRIDE (PMID: 30395289) partner repository with the dataset identifier PXD027352. Proteomics data are additionally provided in Supplementary file 1.

Field-specific reporting

Please select the one below that is the best fit for your research. If you are not sure, read the appropriate sections before making your selection.

☒ Life sciences ☐ Behavioural & social sciences ☐ Ecological, evolutionary & environmental sciences

For a reference copy of the document with all sections, see nature.com/documents/nr-reporting-summary-flat.pdf

Life sciences study design

All studies must disclose on these points even when the disclosure is negative.

Sample size	Sample sizes were determined based on experience from previous work and based on other similar published studies such as Steib et al., Elife 2020 or Wang et al., Elife 2017
Data exclusions	no data were excluded from the analysis
Replication	Quantitative experiments were replicated between 2 and 12 times, with similar outcome. For experiments other than quantitative experiments, all attempts at replication were successful. Protein localization analysis has been repeated at least twice (Fig. 1a-e, Fig. 2b-e, Fig. 3b,c,e Fig. 5i, Supplementary Fig. 1a,b, Supplementary Fig. 2e, Supplementary Fig. 3a,c) with similar outcome. The analysis of CP110 localization in U2OS (Fig. 5b) and RPE1 p53 KO (Fig. 5j) cells and POC5 and HAUS6 localization in untreated mitotic cells (Supplementary Fig. 4a,b) has been performed once. Dependencies have been confirmed in at least two independent experiments (Fig. 2c-g, Fig. 3d, Supplementary Fig. 2a,b,d,f), except for CEP192 localization after CEP192 RNAi (Fig. 2b), which has been analyzed only once. Microtubule regrowth along the centriole wall (Fig. 1b) has been confirmed in at least two independent experiments. The BioID was performed once. Depletion of HAUS6 or POC5 after RNAi (Supplementary Fig. 2c, Supplementary Fig. 3b) or HAUS6 depletion after HAUS6 KO induction (Fig. 5e) has been confirmed at least two times by Western Blot analysis.
Randomization	randomization was not relevant to this study as no animal or human subjects were involved in this study (mice were only used to obtain neuronal cutures).
Blinding	investigators were not blinded to group allocations as groups could be identified by differences in immunofluorescence staining patterns in most experiments. For other experiments, efficiently depleted cells had to be identified prior to analysis as the percentage of those cells within the population was otherwise too low.

Reporting for specific materials, systems and methods

We require information from authors about some types of materials, experimental systems and methods used in many studies. Here, indicate whether each material, system or method listed is relevant to your study. If you are not sure if a list item applies to your research, read the appropriate section before selecting a response.

Materials & experimental systems

n/a	Involved in the study
☐	☒ Antibodies
☐	☒ Eukaryotic cell lines
☒	☐ Palaeontology and archaeology
☐	☒ Animals and other organisms
☒	☐ Human research participants
☒	☐ Clinical data
☒	☐ Dual use research of concern

Methods

n/a	Involved in the study
☒	☐ ChIP-seq
☒	☐ Flow cytometry
☒	☐ MRI-based neuroimaging

Antibodies

Antibodies used	rabbit anti-HAUS6 and rabbit anti-GCP4 were home-made. mouse anti-γ-tubulin (TU-30, Exbio), rabbit anti-γ-tubulin R75 (Julian et al., J Cell Scie 1993), rabbit anti-alpha-tubulin (ab18251, Abcam), mouse anti-acetylated alpha-tubulin (clone 6-11B-1, Merck), mouse anti-polyglutamylated tubulin (GT335, AdipoGen), rabbit anti-NEDD1 (Luders et al., Nat Cell Biol 2006), rabbit anti-HAUS5 (Lawo et al., Curr Biol 2009), rabbit anti-pericentrin (Luders et al., Nat Cell Biol 2006), rabbit anti-GFP (A6455, Invitrogen), chicken anti-GFP (GFP-1020, Aves Labs), mouse anti-centrin 1 (clone 20H5, Millipore), rabbit anti-POC5 (A303-341A, Bethyl Laboratories), mouse anti-SAS-6 (sc-81431, Santa Cruz), mouse anti-centrobin (Ogungbenro et al., J Cell Biol 2018), rabbit anti-ninein (Srsen et al., BMC Cell Biol 2009), rabbit anti-ODF2 (43840, Abcam), mouse anti-ARL13B (sc-515784, Santa Cruz), rabbit anti-CEP192 (Zhu et al., CB 2008), rabbit anti-CP110 (Proteintech 12780-1-AP), rabbit anti-CP110 (unpublished, gift by Andrew Holland), rabbit anti-CEP164 antibody (Proteintech 22227-1-AP), mouse anti-GAPDH (sc-47724, Santa Cruz). Alexa-Fluor-350-, Alexa-Fluor-488-, Alexa-Fluor-568- and Alexa-Fluor-647-conjugated, cross-adsorbed secondary goat anti-rabbit or goat anti-mouse antibodies (Thermo Fisher, A11029, A11034, A21131, A21121, A11036, A11031, A21144, A21068, A21244) . HRP-coupled secondary goat anti-rabbit and goat anti-mouse antibodies (Jackson ImmunoResearch Laboratories, AB_10015289, AB_2313567).
Validation	For commercial/published antibodies, staining patterns in cells matched the expected/published characteristics, band sizes in westernblots were as expected. For some antibodies, including all custom-made antibodies, specificity was confirmed by RNAi or KO (for the anti-HAUS6 antibody e.g. shown by WB in Supplementary Fig. 2c for human U2OS cells or in Supplementary Fig. 2f for murine neurons by IF, for the GCP4 antibody shown by IF in Fig. 4f). The unpublished rabbit anti-CP110 antibody stained the distal end of control centrioles as expected (Fig. 5j), similar to the commercial CP110 antibody (Fig. 5b).

Eukaryotic cell lines

Policy information about cell lines

Cell line source(s)	U2OS, hTERT RPE1 and hTERT BJ cell lines (ATCC), hTERT RPE1 p53 -/- cells (Meng-Fu Bryan Tsou; Wang et al., Elife 2015). Human fibroblasts (Helene Dollfuss; Scheidecker et al., Am. J. Hum. Genet. 2015)
Authentication	Cell lines were not authenticated
Mycoplasma contamination	all cell lines tested negative for mycoplasma contamination
Commonly misidentified lines (See ICLAC register)	no commonly misidentified cell line was used

Animals and other organisms

Policy information about studies involving animals; ARRIVE guidelines recommended for reporting animal research

Laboratory animals	A neuronal specific Haus6 conditional KO mouse strain was generated by crossing Haus6 floxed (Haus6fl) mice (Watanabe et al., Cell Reports 2016; RBRC09630, Accession No. CDB1354K (http://www2.clst.riken.jp/arg/mutant%20mice%20list.html))with B6.Tg(Actl6b-Cre)4092Jiwu/J mice (Jackson Laboratories). Mouse strains were maintained on a mixed C57BL/6 background. Mice used in this study were both male and female. Cell cultures were prepared from e17.5-18.5 mouse embryos.
Wild animals	the study did not involve wild animals
Field-collected samples	the study did not involve samples collected from the field
Ethics oversight	All protocols were approved by the Animal Care and Use Committee of the PCB/University of Barcelona (IACUC; CEEA-PCB) and by the Departament de Territori I Sostenibilitat of the Generalitat de Catalunya in accordance with applicable legislation (Real Decreto 53/2013)

Note that full information on the approval of the study protocol must also be provided in the manuscript.
